# (2*R*,3*S*)-2-Ammonio-3-hydr­oxy-3-(4-nitro­phen­yl)propanoic acid chloride monohydrate

**DOI:** 10.1107/S1600536808012750

**Published:** 2008-05-07

**Authors:** Vincent Gaumet, Valérie Weber, Valery P. Zaitsev, Michel Madesclaire

**Affiliations:** a Laboratoire de Chimie Physique, EA 4231, Faculté de Pharmacie, Université d’Auvergne, 63001 Clermont-Ferrand, France; b Laboratoire de Chimie Thérapeutique, EA 4231, Faculté de Pharmacie, Université d’Auvergne, 63001 Clermont-Ferrand, France; c Chimie Organique, Faculté de Chimie, 443011 Samara, Russian Federation

## Abstract

The title compound, C_9_H_11_N_2_O_5_^+^·Cl^−^·H_2_O, was synthesized from (1*S*,2*S*)-2-amino-1-(4-nitro­phen­yl)propane-1,3-diol in four steps. As demonstrated by this work, no racemization occurs during this synthetic procedure. The crystal structure displays many inter­molecular hydrogen bonds between the acidic cation, chloride anions and the water mol­ecules, forming a three-dimensional network. An intra­molecular bond between the ammonium group and a hydroxyl O atom is also present.

## Related literature

For related compounds see: Crich *et al.* (2007 ▶); Di Giovanni *et al.* (1996 ▶); Easton *et al.* (1996 ▶); Madesclaire *et al.* (2006 ▶, 2007 ▶); Steinreiber *et al.* (2007 ▶); Zaitsev *et al.* (1998 ▶). 
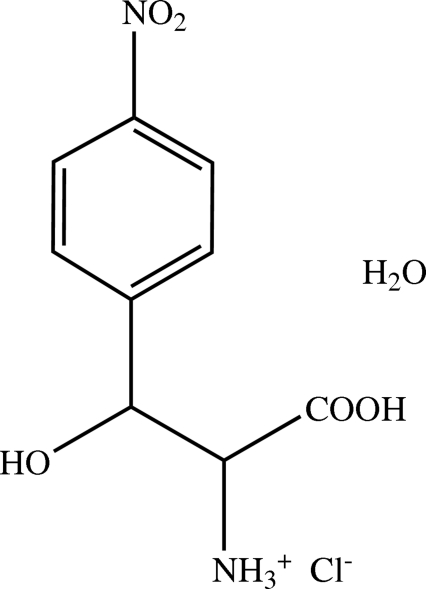


## Experimental

### 

#### Crystal data

C_9_H_11_N_2_O_5_^+^·Cl^−^·H_2_O
*M* *_r_* = 280.66
Monoclinic, 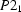

*a* = 8.1286 (17) Å
*b* = 5.056 (3) Å
*c* = 15.848 (3) Å
β = 104.626 (17)°
*V* = 630.2 (4) Å^3^
*Z* = 2
Mo *K*α radiation
μ = 0.33 mm^−1^
*T* = 293 (2) K
0.49 × 0.25 × 0.20 mm

#### Data collection

Enraf–Nonius CAD-4 diffractometer
Absorption correction: ψ scan (North *et al*., 1968 ▶) *T*_min_ = 0.872, *T*_max_ = 0.931
6026 measured reflections
5517 independent reflections
5058 reflections with *I* > 2σ(*I*)
*R*_int_ = 0.021
3 standard reflections every 63 reflections intensity decay: 3%

#### Refinement

*R*[*F* ^2^ > 2σ(*F* ^2^)] = 0.043
*wR*(*F* ^2^) = 0.115
*S* = 1.09
5517 reflections
216 parameters
1 restraint
All H-atom parameters refined
Δρ_max_ = 0.60 e Å^−3^
Δρ_min_ = −0.63 e Å^−3^
Absolute structure: Flack (1983 ▶), 2752 Friedel pairs
Flack parameter: 0.00 (4)

### 

Data collection: *CAD-4 Software* (Enraf–Nonius, 1989 ▶); cell refinement: *CAD-4 Software*; data reduction: *XCAD4* (Harms, 1996 ▶); program(s) used to solve structure: *SHELXS97* (Sheldrick, 2008 ▶); program(s) used to refine structure: *SHELXL97* (Sheldrick, 2008 ▶); molecular graphics: *ORTEP-3* (Farrugia, 1997 ▶) and *PLATON* (Spek, 2003 ▶); software used to prepare material for publication: *SHELXL97*.

## Supplementary Material

Crystal structure: contains datablocks global, I. DOI: 10.1107/S1600536808012750/cs2076sup1.cif

Structure factors: contains datablocks I. DOI: 10.1107/S1600536808012750/cs2076Isup2.hkl

Additional supplementary materials:  crystallographic information; 3D view; checkCIF report

## Figures and Tables

**Table 1:** Hydrogen-bond geometry (Å, °)

*D*—H⋯*A*	*D*—H	H⋯*A*	*D*⋯*A*	*D*—H⋯*A*
N10—H101⋯O13^i^	0.810 (17)	2.420 (16)	2.9259 (13)	121.5 (14)
N10—H101⋯Cl^ii^	0.810 (17)	2.489 (16)	3.2298 (11)	152.7 (15)
N10—H102⋯O14	0.83 (2)	2.265 (19)	2.6759 (14)	110.8 (16)
N10—H102⋯Cl^iii^	0.83 (2)	2.62 (2)	3.3409 (14)	145.5 (16)
N10—H103⋯Cl^iv^	0.97 (3)	2.28 (3)	3.2435 (14)	176 (3)
O12—H12⋯O17^v^	0.78 (5)	1.84 (5)	2.6168 (19)	171 (4)
O14—H14⋯Cl	0.82 (3)	2.25 (3)	3.0539 (13)	166 (3)
O17—H171⋯O16^vi^	0.78 (3)	2.33 (3)	3.069 (2)	159 (4)
O17—H172⋯Cl^vii^	0.76 (3)	2.45 (3)	3.2170 (13)	178 (3)

Symmetry codes: (i) 
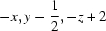
; (ii) 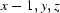; (iii) 
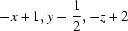
; (iv) 
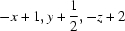
; (v) 
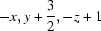
; (vi) 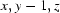; (vii) 
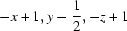
.

**Fig. 1.**
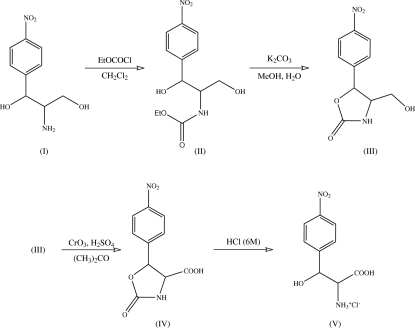
Synthesis of the title compound (V).

**Fig. 2.**
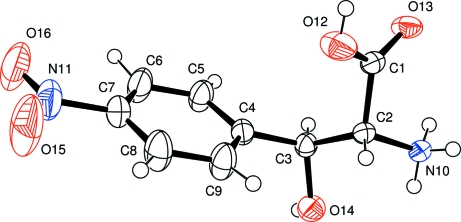
The molecular structure of the title compound (V), with atom labels and 50% probability displacement ellipsoids for non-H atoms.

**Fig. 3.**
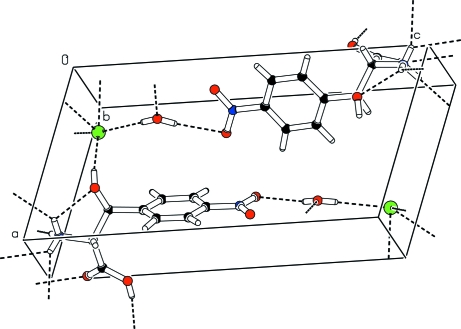
Crystal packing of the title compound. Intra and intermolecular hydrogen bonds are shown as dashed lines. H atoms not involved in the interactions have been omitted.

## supplementary crystallographic information

### Comment

Preparation of new α-amino acids are of constant interest. (1*S*,2*S*) and (1*R*,2*R*)-2-amino-1- (4-nitrophenyl)-1,3-propanediols, byproducts in the manufacture of the antibiotic Chloramphenicol (Zaitsev *et al.*, 1998), can be used as starting materials in the preparation of corresponding α-amino acid isomers. The title compound (V) was synthesized from (1*S*,2*S*)-2-amino-1- (4-nitrophenyl)-1,3-propanediol (I) in four steps according to Figure 1. The intermediate formation of the 2-oxazolidinone derivative (III) allowed to protect both the adjacent amino and hydroxyl groups (Di Giovanni *et al.*, 1996; Crich *et al.*, 2007). The absolute configuration, determined using anomalous dispersion by chlorine, confirms the *R* and *S* configurations respectively for C2 and C3 atoms, confirming that no racemization occurs during this synthetic route (note that the Cahn-Ingold-Prelog designation at the α-carbon of the hydroxyl group is reversed by comparison with that of the starting material due to the change in priority of the substituents). As expected, the phenyl ring is planar, r.m.s. deviation from the best plane is ca. 0.007Å. The nitro group is coplanar with the adjacent phenyl ring (O15—N11—C7—C8 = 0.2 (3)° and O16—N11—C7—C8 = 179.9 (2)°). However, the σ bond C7—N11 (1.4704 (17) Å) shows that there is no appreciable π delocalization in the bond between the *sp*^2^ hybridized N11 and the phenyl ring.

### Experimental

Figure 1 summarizes the synthetic route used (Madesclaire *et al.*, 2006 and 2007). Crystals suitable for an X-ray diffraction study were obtained by slow evaporation of a water solution containing compound (V).

### Refinement

The structure was solved by direct methods and refined with anisotropic temperature factors for non-H atoms. All H atoms were found from difference Fourier maps. The H atoms were all refined isotropically with no constraints.

### Crystal data

**Table:**

C_9_H_11_N_2_O_5_^+^·Cl^–^·H_2_O	*F*_000_ = 292
*M**_r_* = 280.66	*D*_x_ = 1.479 Mg m^−^^3^
Monoclinic, *P*2_1_	Mo *K*α radiation λ = 0.71073 Å
Hall symbol: P 2yb	Cell parameters from 25 reflections
*a* = 8.1286 (17) Å	θ = 8–18º
*b* = 5.056 (3) Å	µ = 0.33 mm^−^^1^
*c* = 15.848 (3) Å	*T* = 293 (2) K
β = 104.626 (17)º	Elongated prism, colourless
*V* = 630.2 (4) Å^3^	0.49 × 0.25 × 0.20 mm
*Z* = 2	

### Data collection

**Table:**

Enraf–Nonius CAD-4 diffractometer	*R*_int_ = 0.022
Radiation source: fine-focus sealed tube	θ_max_ = 35.0º
Monochromator: graphite	θ_min_ = 1.3º
*T* = 293(2) K	*h* = −13→13
ω/2θ scans	*k* = −8→8
Absorption correction: ψ scan(North et al., 1968)	*l* = −25→25
*T*_min_ = 0.872, *T*_max_ = 0.931	3 standard reflections
6026 measured reflections	every 63 reflections
5517 independent reflections	intensity decay: 3%
5058 reflections with *I* > 2σ(*I*)	

### Refinement

**Table:**

Refinement on *F*^2^	Hydrogen site location: inferred from neighbouring sites
Least-squares matrix: full	All H-atom parameters refined
*R*[*F*^2^ > 2σ(*F*^2^)] = 0.043	*w* = 1/[σ^2^(*F*_o_^2^) + (0.0825*P*)^2^ + 0.0202*P*] where *P* = (*F*_o_^2^ + 2*F*_c_^2^)/3
*wR*(*F*^2^) = 0.115	(Δ/σ)_max_ = 0.001
*S* = 1.09	Δρ_max_ = 0.60 e Å^−^^3^
5517 reflections	Δρ_min_ = −0.63 e Å^−^^3^
216 parameters	Extinction correction: SHELXL97 (Sheldrick, 2008), Fc^*^=kFc[1+0.001xFc^2^λ^3^/sin(2θ)]^-1/4^
1 restraint	Extinction coefficient: 0.089 (10)
Primary atom site location: structure-invariant direct methods	Absolute structure: Flack (1983), 2752 Friedel pairs
Secondary atom site location: difference Fourier map	Flack parameter: 0.00 (4)

### Special details

**Table:**

Experimental. North A.C.T., Phillips D.C. & Mathews F.S. (1968) Acta. Cryst. A24, 351. Number of psi-scan sets used was 4. Theta correction was applied. Weighted transmission curves were used. No Fourier smoothing was applied.

### Fractional atomic coordinates and isotropic or equivalent isotropic displacement parameters (Å^2^)

**Table:**

	*x*	*y*	*z*	*U*_iso_*/*U*_eq_	
Cl	0.71520 (3)	0.93514 (6)	0.922526 (17)	0.03591 (8)	
C1	−0.00336 (13)	1.2561 (2)	0.83505 (7)	0.02674 (16)	
C2	0.09722 (12)	1.00092 (18)	0.85613 (6)	0.02405 (15)	
C3	0.27337 (13)	1.0067 (2)	0.83713 (6)	0.02743 (16)	
C4	0.26848 (14)	1.0458 (2)	0.74189 (7)	0.02986 (18)	
C5	0.3717 (2)	1.2362 (3)	0.71889 (9)	0.0427 (3)	
C6	0.3767 (3)	1.2672 (4)	0.63235 (10)	0.0499 (4)	
C7	0.2774 (2)	1.1044 (3)	0.57084 (8)	0.0415 (3)	
C8	0.1743 (2)	0.9107 (4)	0.59137 (9)	0.0506 (4)	
C9	0.1720 (2)	0.8796 (3)	0.67820 (9)	0.0459 (3)	
N10	0.12271 (11)	0.9477 (2)	0.95052 (5)	0.02702 (14)	
N11	0.2786 (2)	1.1354 (4)	0.47874 (9)	0.0569 (4)	
O12	−0.08726 (19)	1.2705 (3)	0.75327 (6)	0.0491 (3)	
O13	−0.00519 (12)	1.42165 (18)	0.88974 (5)	0.03491 (16)	
O14	0.34476 (13)	0.7557 (2)	0.86696 (7)	0.03708 (19)	
O15	0.1882 (3)	0.9890 (7)	0.42545 (9)	0.0966 (9)	
O16	0.3691 (3)	1.3062 (5)	0.45945 (10)	0.0847 (6)	
H2	0.034 (3)	0.872 (5)	0.8304 (13)	0.037 (5)*	
H3	0.343 (3)	1.127 (5)	0.8692 (13)	0.032 (5)*	
H5	0.445 (3)	1.340 (6)	0.7648 (17)	0.052 (6)*	
H6	0.453 (4)	1.424 (10)	0.620 (2)	0.091 (10)*	
H8	0.112 (5)	0.807 (10)	0.548 (2)	0.087 (10)*	
H9	0.090 (4)	0.766 (8)	0.6899 (19)	0.073 (9)*	
H101	0.032 (2)	0.927 (4)	0.9615 (10)	0.025 (3)*	
H102	0.186 (2)	0.816 (4)	0.9639 (12)	0.030 (4)*	
H103	0.174 (4)	1.087 (7)	0.9905 (16)	0.058 (7)*	
H12	−0.147 (5)	1.395 (10)	0.743 (2)	0.087 (10)*	
H14	0.448 (3)	0.778 (6)	0.8856 (15)	0.049 (6)*	
O17	0.29100 (18)	0.1787 (3)	0.26402 (8)	0.0491 (3)	
H171	0.307 (4)	0.250 (8)	0.309 (2)	0.073 (9)*	
H172	0.290 (3)	0.237 (6)	0.2194 (17)	0.053 (7)*	

### Atomic displacement parameters (Å^2^)

**Table:**

	*U*^11^	*U*^22^	*U*^33^	*U*^12^	*U*^13^	*U*^23^
Cl	0.03069 (11)	0.03544 (12)	0.03887 (13)	0.00116 (10)	0.00372 (8)	−0.00215 (10)
C1	0.0319 (4)	0.0221 (3)	0.0267 (4)	0.0005 (3)	0.0084 (3)	0.0022 (3)
C2	0.0281 (3)	0.0200 (3)	0.0249 (3)	−0.0009 (3)	0.0083 (3)	0.0000 (2)
C3	0.0288 (4)	0.0274 (4)	0.0276 (4)	−0.0040 (3)	0.0099 (3)	−0.0011 (3)
C4	0.0339 (4)	0.0297 (4)	0.0287 (4)	−0.0028 (3)	0.0130 (3)	−0.0025 (3)
C5	0.0576 (7)	0.0393 (6)	0.0359 (5)	−0.0182 (6)	0.0206 (5)	−0.0051 (4)
C6	0.0705 (10)	0.0481 (7)	0.0377 (6)	−0.0171 (7)	0.0260 (6)	−0.0004 (5)
C7	0.0531 (7)	0.0457 (7)	0.0295 (5)	0.0025 (5)	0.0176 (5)	0.0005 (4)
C8	0.0595 (8)	0.0634 (10)	0.0304 (5)	−0.0190 (8)	0.0141 (5)	−0.0110 (6)
C9	0.0582 (8)	0.0504 (8)	0.0323 (5)	−0.0227 (6)	0.0172 (5)	−0.0090 (5)
N10	0.0337 (3)	0.0226 (3)	0.0266 (3)	0.0022 (3)	0.0110 (2)	0.0036 (3)
N11	0.0739 (10)	0.0692 (11)	0.0324 (5)	−0.0018 (8)	0.0221 (6)	0.0009 (6)
O12	0.0684 (7)	0.0451 (5)	0.0274 (4)	0.0240 (5)	0.0005 (4)	0.0003 (4)
O13	0.0471 (4)	0.0216 (3)	0.0339 (3)	0.0039 (3)	0.0062 (3)	−0.0026 (3)
O14	0.0310 (4)	0.0371 (4)	0.0441 (5)	0.0061 (3)	0.0112 (3)	0.0058 (4)
O15	0.1309 (16)	0.127 (2)	0.0336 (5)	−0.0504 (17)	0.0250 (8)	−0.0151 (8)
O16	0.1330 (17)	0.0846 (12)	0.0465 (7)	−0.0305 (13)	0.0414 (9)	0.0055 (8)
O17	0.0652 (7)	0.0431 (5)	0.0357 (5)	−0.0145 (5)	0.0070 (4)	0.0004 (4)

### Geometric parameters (Å, °)

**Table:**

C1—O13	1.2078 (14)	C7—C8	1.380 (2)
C1—O12	1.3053 (14)	C7—N11	1.4704 (17)
C1—C2	1.5188 (15)	C8—C9	1.3898 (18)
C2—N10	1.4821 (12)	C8—H8	0.91 (4)
C2—C3	1.5360 (14)	C9—H9	0.94 (4)
C2—H2	0.87 (2)	N10—H101	0.810 (17)
C3—O14	1.4253 (16)	N10—H102	0.83 (2)
C3—C4	1.5128 (14)	N10—H103	0.97 (3)
C3—H3	0.90 (2)	N11—O15	1.219 (3)
C4—C5	1.3851 (17)	N11—O16	1.222 (3)
C4—C9	1.3931 (18)	O12—H12	0.78 (5)
C5—C6	1.3913 (19)	O14—H14	0.82 (3)
C5—H5	0.97 (3)	O17—H171	0.78 (3)
C6—C7	1.372 (2)	O17—H172	0.76 (3)
C6—H6	1.05 (4)		
			
O13—C1—O12	125.15 (11)	C5—C6—H6	115.3 (17)
O13—C1—C2	122.31 (10)	C6—C7—C8	122.79 (12)
O12—C1—C2	112.53 (9)	C6—C7—N11	119.42 (14)
N10—C2—C1	107.85 (8)	C8—C7—N11	117.79 (14)
N10—C2—C3	107.55 (8)	C7—C8—C9	118.33 (14)
C1—C2—C3	114.68 (8)	C7—C8—H8	119 (3)
N10—C2—H2	104.6 (14)	C9—C8—H8	123 (3)
C1—C2—H2	108.4 (15)	C8—C9—C4	120.22 (13)
C3—C2—H2	113.1 (14)	C8—C9—H9	117.0 (19)
O14—C3—C4	110.69 (9)	C4—C9—H9	121.9 (19)
O14—C3—C2	103.87 (8)	C2—N10—H101	109.8 (11)
C4—C3—C2	114.02 (9)	C2—N10—H102	108.8 (13)
O14—C3—H3	105.5 (14)	H101—N10—H102	112 (2)
C4—C3—H3	109.3 (13)	C2—N10—H103	117.1 (17)
C2—C3—H3	113.1 (13)	H101—N10—H103	102 (2)
C5—C4—C9	119.76 (11)	H102—N10—H103	106 (2)
C5—C4—C3	119.24 (10)	O15—N11—O16	123.43 (16)
C9—C4—C3	120.81 (10)	O15—N11—C7	117.97 (18)
C4—C5—C6	120.54 (13)	O16—N11—C7	118.60 (17)
C4—C5—H5	118.4 (17)	C1—O12—H12	113 (2)
C6—C5—H5	120.9 (17)	C3—O14—H14	107 (2)
C7—C6—C5	118.32 (13)	H171—O17—H172	129 (4)
C7—C6—H6	126.2 (17)		
			
O15—N11—C7—C6	−179.5 (2)	O14—C3—C4—C9	62.57 (15)
O16—N11—C7—C6	0.2 (3)	O14—C3—C4—C5	−112.37 (13)
O15—N11—C7—C8	0.2 (3)	C2—C3—C4—C9	−54.08 (14)
O16—N11—C7—C8	179.9 (2)	C5—C4—C9—C8	−2.4 (2)
O12—C1—C2—C3	84.78 (12)	C3—C4—C9—C8	−177.27 (13)
O12—C1—C2—N10	−155.44 (11)	C9—C4—C5—C6	1.6 (2)
O13—C1—C2—N10	23.39 (14)	C3—C4—C5—C6	176.57 (15)
O13—C1—C2—C3	−96.39 (12)	C4—C5—C6—C7	−0.2 (3)
C1—C2—C3—O14	176.50 (9)	C5—C6—C7—N11	179.19 (18)
C1—C2—C3—C4	−62.95 (11)	C5—C6—C7—C8	−0.5 (3)
N10—C2—C3—C4	177.11 (8)	C6—C7—C8—C9	−0.2 (3)
N10—C2—C3—O14	56.56 (10)	N11—C7—C8—C9	−179.96 (17)
C2—C3—C4—C5	130.98 (12)	C7—C8—C9—C4	1.7 (2)

### Hydrogen-bond geometry (Å, °)

**Table:**

*D*—H···*A*	*D*—H	H···*A*	*D*···*A*	*D*—H···*A*
N10—H101···O13^i^	0.810 (17)	2.420 (16)	2.9259 (13)	121.5 (14)
N10—H101···Cl^ii^	0.810 (17)	2.489 (16)	3.2298 (11)	152.7 (15)
N10—H102···O14	0.83 (2)	2.265 (19)	2.6759 (14)	110.8 (16)
N10—H102···Cl^iii^	0.83 (2)	2.62 (2)	3.3409 (14)	145.5 (16)
N10—H103···Cl^iv^	0.97 (3)	2.28 (3)	3.2435 (14)	176 (3)
O12—H12···O17^v^	0.78 (5)	1.84 (5)	2.6168 (19)	171 (4)
O14—H14···Cl	0.82 (3)	2.25 (3)	3.0539 (13)	166 (3)
O17—H171···O16^vi^	0.78 (3)	2.33 (3)	3.069 (2)	159 (4)
O17—H172···Cl^vii^	0.76 (3)	2.45 (3)	3.2170 (13)	178 (3)

Symmetry codes: (i) −*x*, *y*−1/2, −*z*+2; (ii) *x*−1, *y*, *z*;  (iii) −*x*+1, *y*−1/2, −*z*+2; (iv) −*x*+1, *y*+1/2, −*z*+2; (v) −*x*, *y*+3/2, −*z*+1; (vi) *x*, *y*−1, *z*; (vii) −*x*+1, *y*−1/2, −*z*+1.
